# Report of the Signal Transduction Society Meeting 2017—Metabolism in Health and Disease

**DOI:** 10.3390/ijms19020549

**Published:** 2018-02-12

**Authors:** Bastian Schirmer, Klaudia Giehl, Katharina F. Kubatzky

**Affiliations:** 1 Institut für Pharmakologie, Medizinische Hochschule Hannover, Carl-Neuberg-Str. 1, 30625 Hannover, Germany; schirmer.bastian@mh-hannover.de; 2 Signaltransduktion Zellulärer Motilität, Innere Medizin V, Justus-Liebig-Universität Giessen, Aulweg 128, 35392 Giessen, Germany; klaudia.giehl@innere.med.uni-giessen.de; 3 Zentrum für Infektiologie, Medizinische Mikrobiologie und Hygiene, Universitätsklinikum Heidelberg, Im Neuenheimer Feld 324, 69120 Heidelberg, Germany

**Keywords:** signal transduction, STS, conference report, metabolism, receptor signaling, infection and inflammation, cellular motility and cytoskeleton, translational cancer research, growth factors, cytokines

## Abstract

The annual “Joint Meeting Signal Transduction—Receptors, Mediators and Genes” of the Signal Transduction Society (STS) aims to be an interdisciplinary forum for researchers who share a common interest in deciphering signal transduction pathways in normal or transformed cells, in health and disease, in humans and animal models, or in plants or bacteria. The special focus of the 21st annual Joint Meeting, which took place from 8–10 November 2017 in Weimar, was the topic “Metabolism in Health and Disease” and covered multiple aspects of this highly exciting and fast developing research field. Invited keynote speakers introduced the impact of metabolism on tumor immunology, immune cell signaling, and posttranslational modifications in three specific workshops to the audience. Various other aspects of signal transduction were intensively discussed in five additional workshops. Here, we give an overview of the various workshops and further aspects of the scientific program.

## 1. Introduction

The annual Joint Meeting “Signal Transduction—Receptors, Mediators and Genes” took place in Weimar, Germany, from 8 to 10 November 2017 [1]. The conference was organized by the Signal Transduction Society (STS) together with signaling study groups of the German Societies for Cell Biology (DGZ), for Biochemistry and Molecular Biology (GBM), for Immunology (DGfI), and for Pharmacology (DGP). Moreover, the interdisciplinary aspect of the meeting was strengthened by the support from the study group Infection Immunology of the DGfI and the German Society for Hygiene and Medical Microbiology (DGHM), the SFB 854 (Collaborative Research Centre 854) “Molecular Organisation of Cellular Communication in the Immune System” (B. Schraven, Magdeburg) as well as the graduate school 2155 ProMoAge (A. Simm, Jena/Halle), the TRR 130 (Transregional Collaborative Research Centre 130) “B Cell Immunity and Autoimmunity” (L. Nitschke, Erlangen) and the DGfI study group “Biology of B lymphocytes” (J. Wienands, Göttingen) that contributed scientifically and financially to the program. The meeting organization was performed by the STS council together with the chairpersons of the study groups and the members of the STS Advisory Board. The highly topical focus “Metabolism in Health and Disease” was coordinated by the DGfI study group Signal Transduction with a number of exciting keynote lectures.

## 2. Meeting Overview

### 2.1. Focus Topic

While the groundwork of deciphering the cellular metabolism was already done in the early 20th century, the field currently experiences a renaissance as it becomes clear that changes in cellular activity provide clues not only as to how diseases evoke and progress, but also about cellular regulation, activation, or differentiation [2].

The first of the three focus workshops was entitled “Metabolism and Tumour Immunology”, and the keynote presentation by Dirk Brenner (Luxembourg) highlighted that reactive oxygen species (ROSs) are more than a simple by-product of mitochondrial metabolism. In fact, ROSs can act as signaling molecules that are needed for fighting viral infections and might also play a role in autoimmunity. Activated T-cells show an enhanced ROS production and the subsequent upregulation of the glutathione (GSH)-producing glutamate-cysteine ligase provides a response mechanism that allows maximal activation without cell damage. Inhibition of GSH production in a mouse model therefore reduced the ability of the activated T-cells to switch their metabolism to glycolysis to satisfy short-term energy demands. This led to an impaired antiviral response, but also protected from autoimmune disease [3]. In the second keynote presentation of this workshop, Luciana Berod (Hannover, Germany) showed that the dynamic regulation of metabolic processes can shape T-cell response as well as T-cell differentiation. Inhibition of lipid synthesis through the Myxobacterium-derived macrolide Soraphen A triggers the differentiation into regulatory T cells. In a genetic approach, the T-cell-specific ablation of the Acetyl-CoA Carboxylase (ACC1) inhibited T-cell differentiation, while the differentiation into Tregs remained unchanged, suggesting that fatty acid oxidation controls the balance between effector cells and regulatory cells [4,5]. Three short talks chosen from the submitted abstracts completed the workshop.

The second focus workshop “Metabolism and Immune Cell Signalling” started with a presentation by Michael R. Gold (Vancouver, BC, Canada) who explained why B-cell receptor (BCR) signaling processes need to be investigated at the nanoscale. Not only does the composition of plasma membranes vary between cell types, but there is a much higher degree of membrane organization than previously thought. Using super-resolution microscopy and proximity ligation assays (PLAs), it is possible to investigate signaling events at the plasma membrane at nanometer distances. Prof. Gold showed that the BCR form so-called nanoclusters that, upon activation, can reorganize into microclusters through actin-mediated events thereby recruiting further signaling molecules. He also nicely demonstrated how actin dynamics affects BCR signaling and the immune synapse [6,7]. Further aspects of B-cell and T-cell signaling were also addressed in the following 15 min short talks.

The third focus workshop introduced the field of “Metabolism and Posttranslational Modifications” to the audience. Here, Gerald W. Hart (Baltimore, MD, USA) focused on a protein modification that is less well-known, namely the *O*-glycosidic linkage between *N*-acetylglucosamine (*O*-GlcNAc) and serine or threonine residues of nucleocytoplasmic proteins. *O*-GlcNAc cycles on and off nearly all proteins involved in transcription and acts as a sensor that regulates gene transcription depending on the cell’s nutrient supply [8]. However, he showed that this molecule can also act as a signal to regulate nutrient intake, as genetic deletion of *O*-GlcNAc-transferase resulted in an obese mouse with defective satiety. Because *O*-GlcNAc can be found on 50% of all human kinases and *O*-GlcNAc cycling is elevated in many cancers, this modification might represent an interesting drug target [9,10]. As posttranslational modifications are crucial for many aspects in health and disease, different modifications and their impact were discussed in five additional short talks.

### 2.2. Further Keynote Lectures

Receptor signaling is a key aspect in signal transduction and cellular homeostasis. Therefore, the meeting started with a workshop on “Receptor Signalling” and a keynote talk given by Liam O’Mahony (Zurich, Switzerland). Prof. O’Mahony described how bacteria can influence host immunological processes through the production of histamine. Histamine is known to modulate the host immune response in a variety of disease states, but especially in allergic conditions. Thus, the gut microbiota or rather the balance between histamine producing and degrading bacteria may influence the outcome of, e.g., asthma patients and pose a new therapeutic challenge. As presented in his talk, a significant overrepresentation of histamine secreting bacteria could be detected in obese patients, but not in patients of normal weight, implying that an effective therapy may also have to take the gut microbiota into account [11,12].

The workshop on “Infection and Inflammation” was started off by Christina L. Stallings (St. Louis, MO, USA) who gave an inspiring talk on the immune responses to *Mycobacterium tuberculosis* (Mtb). Her talk showed that neutrophils are more than “live fast, die early” cells. They are indeed important factors in establishing a replication niche for Mtb and that this is dependent on the bacterial-induced inhibition of macro-autophagy processes in neutrophils. While a stimulation of autophagy increases clearance, the inhibition of autophagy and specifically Atg5 generates a replication niche for Mtb [13,14]. It will be very interesting to analyze these autophagy-dependent and -independent mechanisms in more detail.

Laura M. Machesky (Glasgow, UK) opened the workshop on “Cellular Motility and Cytoskeleton” with a movie showing how an ameba chases yeast cells to illustrate her research topic that aims at understanding the regulation of polarized cell migration and invasion. Since both of these processes are important factors in cancer metastasis, a detailed molecular understanding is needed. Prof. Machesky showed that the small GTPase Rac1 plays a central role in this process but stated that—despite the many details that are already known on specific pathways involved in cell migration and invasion—information on the inter-connection of these signaling pathways is still scarce. In her talk, she concentrated on the importance of the Rac1–Scar/Wave and Arp2/3 axis for pseudopodia and lamellipodia formation and how this complex is regulated by multiple binding partners by discussing yet unpublished data.

The workshop “Growth Factors and Cytokines” was introduced by the chemist and structural biologist Thomas Müller (Würzburg, Germany), who elaborated on the importance of high-resolution structure details for the design of novel cytokine antagonists. The cytokines interleukin-4 (IL-4) and the related IL-13 and IL-5 are crucial factors in the pathogenesis of atopic diseases and can trigger allergic hypersensitivity reactions. This makes them interesting targets for pharmacological intervention, for example by generating neutralising antibodies. Prof. Müller’s laboratory has employed a combination of mutational studies to change posttranslational modifications, binding studies, and cell-based assays to increase effector affinity and stability and thereby succeeded in generating antibodies with enhanced neutralizing activity (further reading: [15]).

The pathway that is probably most closely connected to tumor biology and tumorigenesis is the PI3K-Akt-mTOR pathway. Manfred Jücker (Hamburg, Germany) presented his work on PI3K-Akt-mTOR signaling in the workshop on “Translational Cancer Research” and provided an inspiring overview of this topic. Despite the central role of mTOR in cancer cell metabolism, emphasized by the frequent finding of constitutively activated mTOR signaling in various human cancers, monotherapies targeting mTOR have failed. Prof. Jücker highlighted feedback loops within the PI3K-Akt-mTOR axis, where for example the downregulation of mTOR results in the upregulation of PI3K and Akt, thereby counteracting mTOR inhibition. Indeed, combined therapies with inhibitors of both mTOR and Akt showed promising results in in vivo studies in mice [16,17]. Finally, the session was concluded by the keynote talk of Almut Schulze (Würzburg, Germany) who pointed out that finding metabolic vulnerabilities of cancer cells might provide a rationale for targeting metabolic processes as therapeutic options. As cancer cells have a high proliferation rate and an increased demand for nutrients and building blocks, they reprogram their metabolism pathways according to their needs. However, this also renders them more vulnerable to certain metabolic changes, especially during stress situations where supply with oxygen or nutrients is low. Metabolic profiling of cancer cells thus allows for the identification of particularly interesting hotspots that might have the potential to serve as therapeutic targets [18,19].

All of these keynote lectures in the different workshops were again followed by a number of selected short talks chosen from the submitted abstracts. As every year, the stimulating mixture of presentations given by group leaders, post-doctoral fellows, and PhDs was an outstanding feature of the STS meeting.

## 3. The STS Medal Award

Since 2010, the STS honors an exceptional scientist in the field of signal transduction research. The award session is concluded by the “Honorary Medal Lecture” given by the awardee. The Honorary Medal was initially introduced by the STS in cooperation with the open access journal “Cell Communication and Signalling” (CCS). This year, the award was co-sponsored for the first time by the publishing group MDPI, specifically the International Journal of Molecular Sciences (IJMS). Previous award winners were Tony Pawson, Tony Hunter, Carl-Henrik Heldin, Klaus Rajewsky, the Nobel Prize winner Jules Hoffmann, Mina Bissell, and Tak Wah Mak. In light of these well-known international scientists, the STS was especially proud to honor Prof. Michael Reth (Freiburg, Germany), a German scientist and long-time STS member. Prof. Reth received the 2017 STS Honorary medal for his lifetime contributions on intracellular signaling pathways in lymphocytes and his pioneering work on the structure and function of the B-cell receptor. Not only did Prof. Reth identify the ITAMs as decisive signaling modules within immune receptors [20], he also developed new techniques for analyzing the nanoscale organization of cell surface proteins [21,22]. Moreover, he applied innovative approaches of synthetic biology to define mechanistic aspects of key signaling processes, leading to a new model of B-cell activation [23]. His ideas and work profoundly influenced and shaped our current understanding of immunity and lymphoma development. The laudatio was given by Jürgen Wienands (Göttingen, Germany), who had been a group leader in Michael Reth’s laboratory, and Michael Gold, a long-time competitor, collaborator, and close friend of Prof. Reth. In his Honorary Medal Lecture, Prof. Reth covered the historical development of our modern model of B-cell receptor complex assembly, BCR signaling strategies and the use of super-resolution techniques to explore the nanoworld of BCR signaling. His lecture was very much appreciated by the audience and was followed by a lively discussion.

## 4. Focus on Early Career Researchers

The support of young scientists has always been a key aspect of the STS Joint Meetings. The 2017 STS Science Award of €1500 was again co-sponsored by BIOMOL GmbH. Since 2005, this prize honors outstanding research by a post-doctoral researcher or a junior principal investigator of the STS. Julia Jellusova (Freiburg, Germany) and Manoj Balakrishna Menon (Hannover, Germany) were awarded the STS Science Award jointly for their individual scientific work and the presentation of their data at the meeting.

At the occasion of the 21st meeting, the STS grant committee also selected 10 students currently working on their Master, MD, or PhD thesis to receive travel grants of €2500 in total to support their meeting attendance. Moreover, all participants had the opportunity to present their poster work during the well-known “one minute–one transparency” session to attract the audience to the following extensive poster discussion in a casual atmosphere. Five poster award winners were selected from about 50 poster presentations and rewarded with a total of €750 of prize money.

## 5. Final Remarks

In the meantime, preparations for the 22nd STS Joint Meeting have already started. The upcoming meeting is scheduled for 5–7 November 2018 and will again take place at the Leonardo Hotel in Weimar. Since it will also be the 20th jubilee of the STS as a scientific society, the 2018 meeting will cover the past and the future of signal transduction with central findings of the last two decades as well as an outlook on new concepts. Details and updated meeting information can be found at http://www.sigtrans.de and on the STS Facebook account.
